# Uterine Tumor Resembling Ovarian Sex Cord Tumors Type II with Vaginal Vault Recurrence

**DOI:** 10.1155/2019/5231219

**Published:** 2019-11-27

**Authors:** Oriana Marrucci, Paola Nicoletti, Alessandro Mauriello, Simone Facchetti, Lodovico Patrizi, Carlo Ticconi, Francesco Sesti, Emilio Piccione

**Affiliations:** ^1^Department of Surgical Sciences, Section of Gynecology and Obstetrics, University of Rome Tor Vergata, Italy; ^2^Anatomic Pathology, Department of Experimental Medicine, University of Rome Tor Vergata, Italy

## Abstract

UTROSCTs (*Uterine Tumors Resembling Ovarian Sex Cord Tumors)* are rare neoplasms of unknown etiology usually occurring in middle-aged women. Less than 100 cases of UTROSCT have been reported so far. Although the typical behavior of UTROSCT is benign, metastatic and recurrent cases can occur. Here we describe an extremely rare case of vaginal vault recurrence of UTROSCT occurring 5 years after total hysterectomy with bilateral salpingo-oophorectomy. Though rare, UTROSCT should always be taken into account in the differential diagnosis of uterine masses initially considered leiomyomas.

## 1. Introduction

Uterine Tumors Resembling Ovarian Sex Cord Tumors (UTROSCTs) are rare neoplasms of unknown etiology [1]. They usually occur in middle-aged women. Most UTROSCTs behave as tumors of low malignant potential. Clinical characteristics are abnormal uterine bleeding and/or abdominal pain, along with an enlarged uterus or a palpable uterine mass without specific imaging finding [2]. Usually the diagnosis is incidental, following immunohistochemical and ultrastructural studies on the surgical specimen. UTROSCTs have a variable immunohistochemical profile with expression of sex cords, epithelial, and smooth muscle lineages markers. Hysterectomy with or without bilateral salpingo-oophorectomy is usually the treatment for UTROSCT. Here we report a rare case of vaginal vault recurrence (VVR) occurring 5 years after surgery in a woman who underwent hysterectomy with bilateral salpingo-oophorectomy for UTROSCT.

## 2. Case Presentation

In January 2013, a 54-year-old pluriparous woman was admitted to the Department of Obstetrics and Gynecology of Tor Vergata University Hospital (Rome) for recurrent postmenopausal abnormal uterine bleeding. At pelvic examination the uterine volume appeared increased by three times the standard volume.

**Figure 1:**
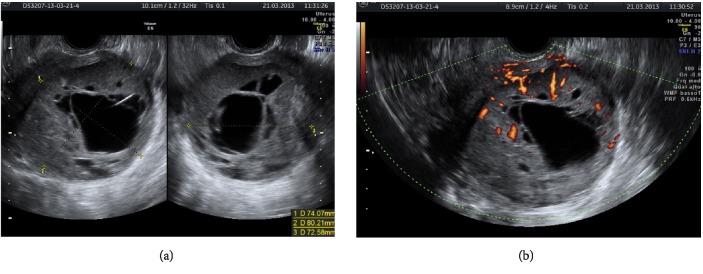
(a) M-Mode findings: enlarged uterus with inhomogeneous echogenicity. (b) Power Doppler findings: multiple anechoic areas with central and peripheral vascularization.

Pelvic transvaginal ultrasound showed an intracavitary mass (80x73x74 mm) with heterogeneous echogenicity in which multiple anechoic areas with central and peripheral vascularization at power Doppler (RI 0.49) were present. The initial diagnosis was uterine leiomyoma. Other intramural leiomyomas, measuring 31x20x31 mm, 18x8 mm, and 9x6 mm, respectively, were identified. Ovaries appeared normal (see Figures 1(a) and 1(b)).

To exclude endometrial origin of bleeding, an office hysteroscopy was performed which was normal.

**Figure 2:**
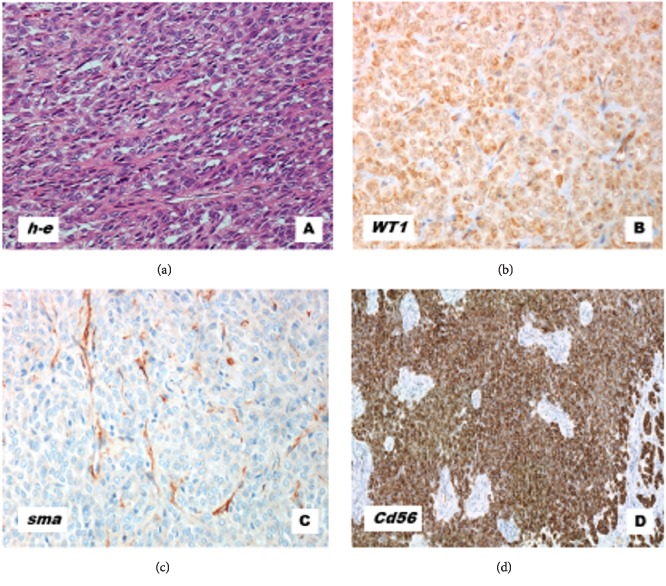
Uterine tumor resembling an ovarian sex cord tumor. (a) Cords, nests, and trabeculae are seen, reminiscent of sex cord cells tumor of the ovary (H-E, 10x). (b) Positive stain for WT1 (10x). (c) Negative stain for smooth muscle actin (SMA, 10x). (d) Positive stain for CD56, a sex cord marker (10x).

Laboratory blood tests showed no significant abnormalities. Total laparoscopic hysterectomy with bilateral salpingo-oophorectomy was performed. At gross pathologic examination, the uterus weighed 359 g and its dimensions were 8x3x4 cm. The uterine surface was deformed due to the presence of a grayish-yellow, polycyclic lesion measuring approximately 9 cm with a soft and fibrous consistency; the lesion also appeared poorly delineated with respect to the surrounding myometrium. On microscopic examination, it was composed of elements similar to the sex cords cells, with a trabecular and alveolar architectural pattern, and tubular elements. The cells showed an epithelioid appearance with irregular nuclei. The mitotic index was equal to 3 mitoses for 10 HPF assessed by means of Ki67 index, whereas proliferation index was 30%. Immunohistochemical profile showed the following: vimentin +, CD 56 +, CD 99 +, WT1 + / -, alpha actin -, desmin -, panCK -, EMA -, CD10 -, caldesmon -, S100 -, inhibin -, CroA -, and synaptophysin -. The diagnosis was uterine tumor with elements similar to the sex cords type II according to Blaustein's 2011 (UTROSCT, according to WHO) [3] (Figure 2).

**Figure 3:**
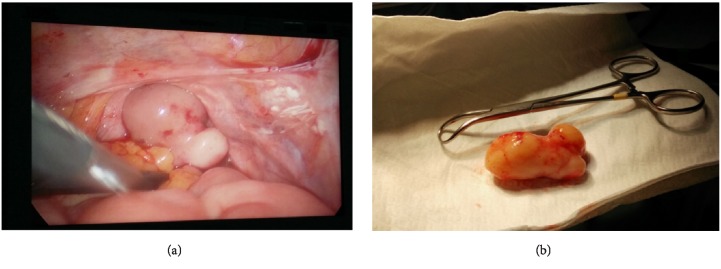
(a) Laparoscopic appearance of the vaginal vault. (b) Macroscopic aspect of the excised mass.

The tubes and ovaries were histologically normal. At regular annual checks the patient remained asymptomatic with normal pelvic examination and ultrasound findings until December 2017 when a transvaginal ultrasound described the presence of a new solid lesion at level of the vaginal vault. The lesion was 45x19x20mm, highly vascularized (color score 4) and had a characteristic arboriform vascular pattern. It was adherent to the surrounding tissues but not to the bladder and bowel wall. These findings were confirmed by a CT scan of the abdomen and pelvis. A laparoscopy was performed with removal of the mass and partial infracolic omentectomy; multiple peritoneal biopsies were also taken (Figure 3(a)). Gross examination of the mass confirmed disease recurrence (Figure 3(b)).

At microscopic examination, the lesion showed elements similar to the sex cords cells, with trabecular and alveolar architecture, and tubular elements. The mitotic index was equal to 3 mitoses for 10 HPF assessed by means of Ki67 index, and proliferation index was 30%. Immunohistochemical profile showed the following: CD 56 +, CD 99 +/-, ER +, WT1 +, alpha actin -, panCK -, EMA -, CD10 -, S100 -, inhibin -, CroA -, calretinin-. The neoplasm was completely removed with free margins. It was compatible with the diagnosis of uterine tumor with elements similar to the sex cords type II according to Blaustein's 2011 (UTROSCT, according to WHO). The morphological and immunohistochemical characteristics were similar to those of the previous uterine neoplasm. No adjuvant therapy was administered according to the oncologic counselling. To date, the patient is asymptomatic, and pelvic examination and ultrasound are normal.

## 3. Discussion

To date, less than 100 cases of UTROSCT have been reported [4]. Although the typical behavior of UTROSCT is benign, metastatic and recurrent cases could occur [5]. To our knowledge, at present only 9 cases with extrauterine spread, distant metastatic disease, or recurrent disease have been reported [6].

UTROSCT is a rare and intriguing tumor. This neoplasm resembles an ovarian sex cord tumor. Even though several hypotheses have been raised to explain the histogenesis of UTROSCT—including its origin from sex cord cells, mesenchymal stem cells, and endometrial stromal tumors in which an excessive growth of sex cord cells occurs [7]—its pathogenesis is still unknown. In 1976, Clement and Scully [8] classified the neoplasm into two distinct subgroups based on clinical and histopathologic features. In the first group, the tumors showed a predominant endometrial stromal tumor-like differentiation and focal ovarian sex-cord-like areas (<50%) and were termed endometrial stromal tumors with sex-cord-like elements (ESTSCLE), whereas the neoplasms in the second group were composed predominantly of a sex-cord-like component accounting for 50% to 100% of the tumor, which was therefore called UTROSCT. UTROSCT has a high variability in the overall architecture of the tumor, as well as in its morphologic features and immunohistochemical characteristics. It shows a great variety of growth patterns, in which the cells are variably organized in sheets, cords, nests, trabeculae, and solid and hollow tubules. The neoplastic cells characteristically show low-grade atypia with round to oval nuclei, with regularly dispersed chromatin and small unnoticeable nucleoli. The cytoplasm is usually scarce and lightly eosinophilic [1]. A plethora of different immunohistochemical markers have been detected in UTROSCT. Some of these markers are (a) sex cord markers: *α*-Inhibin, calretinin, CD56, melan A, CD99, WT1, and MART-1; (b) epithelial markers: pancytokeratin, AE1/3, and epithelial membrane antigen; (c) myoid markers: *α*-smooth muscle actin, desmin, h-caldesmon, and histone-8 deacetylase; (d) neuroendocrine markers: estrogen receptors, progesterone receptors, androgen-receptors, and chromogranin; (e) miscellaneous markers: CD10, S100, CD117, and vimentin [1, 9]. These markers can be variably detected, often coexpressed, and, wherever expressed, can be expressed to a variable extent.

Immunoexpression for calretinin and at least for one of the other sex cord markers above reported is believed to be required to establish a diagnosis of UTROSCT [10]. Hysterectomy with or without bilateral salpingo-oophorectomy is the treatment of choice for UTROSCT [11]. At present, it is still impossible to predict aggressive disease. Traditional high-risk features of uterine cancer are of unclear value in UTROSCT. In UTROSCT lymphovascular space invasion and myometrial invasion have been reported in 4 out of 8 cases in which recurrence occurred [6]. These features have not been found in the present case, in which the initial clinical and US features suggested multiple uterine leiomyomas. Though very rare, UTROSCT should always be taken into account in the differential diagnosis of uterine masses initially considered leiomyomas. Although not specific, US and color Doppler findings could be helpful. There is no available information as to whether adjuvant therapy should be prescribed when VVR of UTROSCT occurs.

To our knowledge, the present case is the tenth one in which VVR of UTROSCT has been described. The possibility of VVR should be considered in the long-term follow-up of patients with UTROSCT, even if they are completely asymptomatic.
