# Erratum to “Uterine Tumor Resembling Ovarian Sex Cord Tumors Type II with Vaginal Vault Recurrence”

**DOI:** 10.1155/2020/5205723

**Published:** 2020-08-19

**Authors:** Oriana Marrucci, Paola Nicoletti, Alessandro Mauriello, Simone Facchetti, Adalgisa Pietropolli, Lodovico Patrizi, Carlo Ticconi, Francesco Sesti, Emilio Piccione

**Affiliations:** ^1^Department of Surgical Sciences Section of Gynecology and Obstetrics, University of Rome Tor Vergata, Italy; ^2^Anatomic Pathology, Department of Experimental Medicine, University of Rome Tor Vergata, Italy

In the article titled “Uterine Tumor Resembling Ovarian Sex Cord Tumors Type II with Vaginal Vault Recurrence” [1], there is an author name “Adalgisa Pietropolli” missing so that the author order in the author list was incorrect, where Oriana Marrucci,^1^ Paola Nicoletti,^1^ Alessandro Mauriello,^2^ Simone Facchetti,^2^ Lodovico Patrizi,^1^ Carlo Ticconi,^1^ Francesco Sesti,^1^ and Emilio Piccione^1^ should have read Oriana Marrucci,^1^ Paola Nicoletti,^1^ Alessandro Mauriello,^2^ Simone Facchetti,^2^ Adalgisa Pietropolli,^1^ Lodovico Patrizi,^1^ Carlo Ticconi,^1^ Francesco Sesti,^1^ and Emilio Piccione^1^.

The correct author order is also shown above in the author information.
